# A descriptive guide for absolute quantification of produced shRNA pseudotyped lentiviral particles by real-time PCR

**DOI:** 10.14440/jbm.2016.142

**Published:** 2016-10-04

**Authors:** Virginie Mournetas, Sofia Melo Pereira, David G. Fernig, Patricia Murray

**Affiliations:** ^1^Department of Cellular and Molecular Physiology, Institute of Translational Medicine, University of Liverpool, L697 ZB Liverpool, United Kingdom; ^2^Department of Biochemistry, Institute of Integrative Biology, University of Liverpool, L697 ZB Liverpool, United Kingdom; ^3^CECS / ISTEM, 2 Rue Henri Desbruères, 91100 Corbeil-Essonnes, France

**Keywords:** lentiviral particle titer, absolute quantification, shRNA, the RNAi Consortium, RT-qPCR

## Abstract

Gene silencing techniques, including RNA interference methodologies, are widely used in reverse genetics to study the role of specific genes in biological processes. RNA interference has become easier to implement thanks to the RNAi Consortium (TRC), which has developed libraries of short hairpin RNA (shRNA) sequences in pseudotyped lentiviral particles capable of targeting most genes in the human and mouse genomes. However, a problem is the lack of a simple method to titrate the homemade lentiviral particle product, making it difficult to optimize and standardize shRNA experiments. Here we provide a guide describing a quick, non-laborious and reliable method for the titration of TRC pseudotyped lentiviral particles that is based on the detection and measurement of viral RNA using quantitative PCR. Our data demonstrate that purified linearized shRNA plasmids represent more suitable standards than circular or unpurified linearized plasmids. We also show that for precise absolute quantification, it is important to determine suitable plasmid and viral cDNA concentrations in order to find the linear range for quantification, as well as to reduce inhibition and primer dimer amplification. Finally, we show that the lentivirus concentration impacts the level of knockdown in transduced cells. Primers utilized in this non-functional titration can potentially be applied to functional titration of proviral DNA copies or transgene expression, overcoming problems arising from the absence of fluorescent reporter genes in TRC plasmids.

## BACKGROUND

The RNAi Consortium (TRC) has developed genome-scale short hairpin (sh)RNA libraries targeting human and mouse genes [1], facilitating lentiviral-based RNA interference (RNAi). Quantification of lentiviruses is important for developing robust and reproducible RNAi protocols. Two types of methods have been elaborated to quantify viral titers: functional titrations, determining the concentration of viral particles needed to transduce a cell; non-functional titrations, determining the number of viral particles secreted by producer cells.

In the context of functional titration, if a fluorescent reporter gene, *e.g.*, green fluorescent protein [2], is transduced alongside the sequence of interest, the functional titer can be determined by flow cytometry. However, this requires a fluorescent marker and cannot distinguish single from multiple proviral DNA integration sites within the host genome. An alternative is to estimate the number of integrated proviral DNA copies per cell by quantitative (q)PCR [2]. Because proviral copies can be inserted into different chromatin regions, the transgene expression can vary [2]. Quantification of the expressed transgene by reverse transcription (RT)-qPCR instead of the proviral DNA copies gives a closer estimation of the transgene expression [2].

Functional titrations require transduction, which is time-consuming, and efficiencies depend on cell type and transgenes [2,3]. Non-functional approaches thus present an easier alternative for determining the titer of a lentiviral particle batch for optimization and standardization. These methods mostly rely on the measurement of either the expressed viral protein p24 or of viral RNA. p24 measured by enzyme-linked immunosorbent assay has reliability issues, due to the presence of a variable amount of p24 originating from defective particles and non-particle-associated p24 [4]. Alternatively, several groups have measured viral RNA, either directly by qPCR [5] or indirectly by RT-qPCR [2,4,6,7]. Even if viral RNA-based titrations also measure defective particles to some extent [6], it has been shown that mainly full-length viral RNA transcripts are incorporated into pseudotyped lentiviral particles [4]. To our knowledge, only one study has described PCR primers suitable for TRC plasmid quantification [7]. However, these primers were within the lentiviral capsid sequence, which is not integrated into the host genome after transduction, limiting their use to non-functional titration.

**Figure 1.**
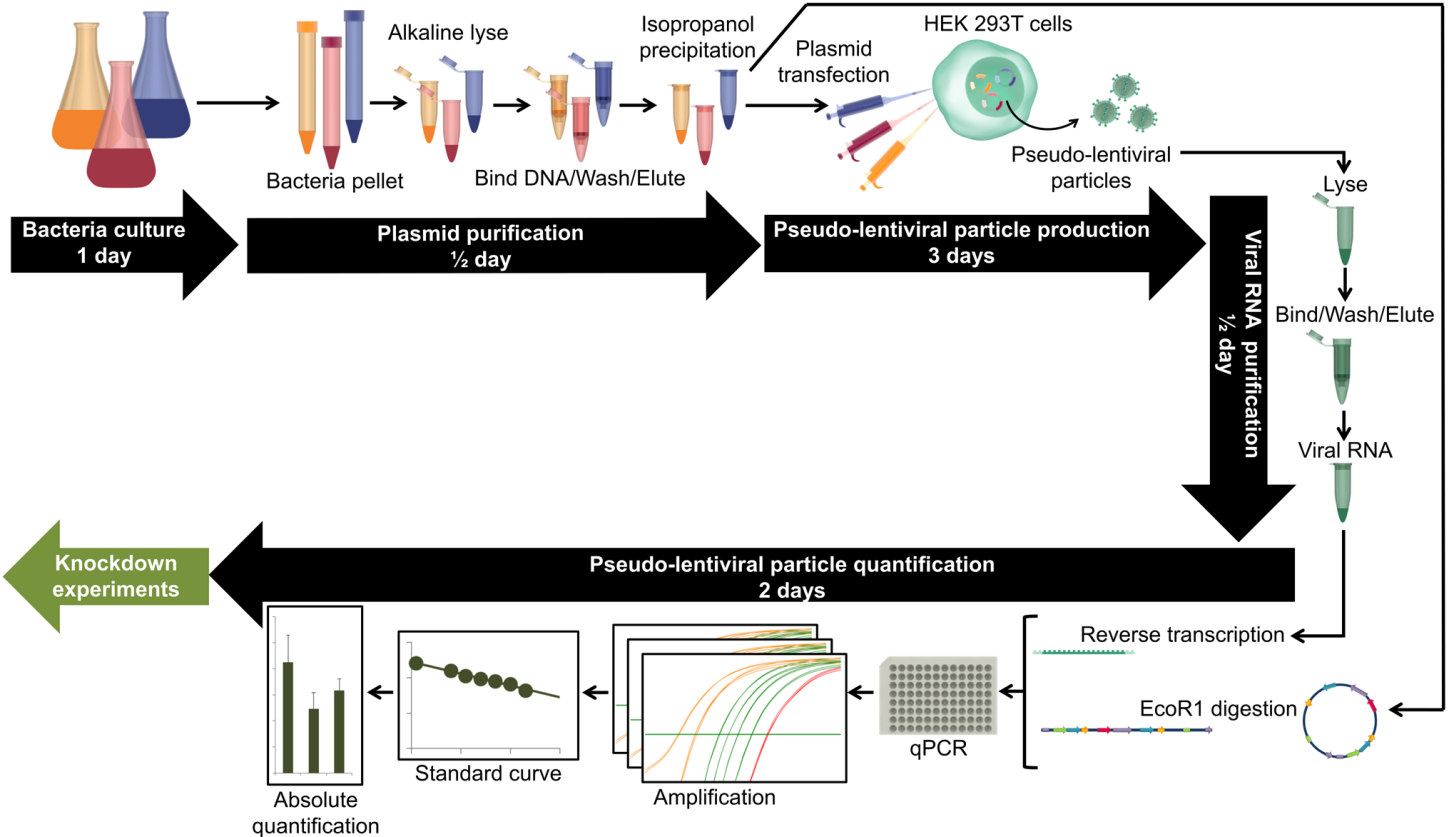
**From plasmid amplification to pseudotyped lentiviral particle quantification.** Main steps in the timeline of pseudotyped lentiviral particle production, starting with plasmid amplification and ending with pseudotyped lentiviral particle absolute quantification.

Here we develop a descriptive guide for absolute titration of homemade TRC shRNA pseudotyped lentiviruses by RT-qPCR using TRC shRNA plasmids as standards. Plasmid amplification and purification followed by pseudotyped lentiviral production take less than 5 d, and then 2 d are needed to determine absolute titers (**Fig. 1**).

We demonstrate that the conformation of the plasmid and its purity (circular, linearized or purified linearized) affect qPCR efficiency, and that absolute quantification requires both the plasmid and viral cDNAs to be within an appropriate concentration range. Designed PCR primers are suitable for functional titration *via* the quantification of integrated proviral DNA copies and/or expressed transgene. Moreover, we show that lentivirus concentration correlates positively with the level of knockdown in transduced cells.

## MATERIALS

### Cells

• Bacteria: *E. coli* containing TRC shRNA plasmids [1] (Sigma-Aldrich Mission shRNA SHCLNG, Dorset UK)
• Producer cells: Human embryonic kidney (HEK)-293TN cells (Cambridge Bioscience LV900A-1, Cambridge UK)
• Target cells: HUES7 line (human embryonic stem cells (hESCs), Harvard University, hES Cell Facility/Melton Laboratory, MA, USA)

### Reagents

• 0.45 μm syringe filters (Fisher Scientific, cat. # 10460031, Loughborough UK)
• Activin A (R&D Systems, cat. # 338-AC-050, Abingdon UK)
• Agarose (Sigma-Aldrich, cat. # A9539, Dorset UK)
• Ampicillin (Sigma-Aldrich, cat. # A5354, Dorset UK)
• β-mercaptoethanol (Invitrogen, cat. # 31350-010, Paisley UK)
• B27 serum free supplement (Invitrogen, cat. # 17504-044, Paisley UK)
• Bovine serum albumin (BSA, Sigma-Aldrich, cat. # A1470-25G, Dorset UK)
• Calcium Phosphate Transfection kit (Sigma-Aldrich, cat. # CAPHOS, Dorset UK)
• Culture dishes (such as 6-well plates, Corning, cat. # 3506, Flintshire UK)
• Distilled nuclease-free water (GIBCO, cat. # 10977-035, Paisley UK)
• DMEM:F12 (Lonza, cat. # BE12-719F, Cambridge UK)
• dNTPs (Invitrogen, cat. # 10297, Paisley UK)
• Dry ice
• EcoR1 enzyme (New England Biolabs, cat. # R3101S, Herts UK)
• Envelope vector pM2D.G (Addgene, cat. # 12259, Middlesex UK)
• Ethanol (Sigma-Aldrich, cat. # 7148, Dorset UK)
• FGF2 (prepared in house [8])
• Fibronectin (EMD Millipore, cat. # FC010-10MG, Watford UK)
• Foetal bovine serum (FBS, GIBCO, cat. # 10270-106, Paisley UK)
• HyperLadder I (Bioline, cat. # BIO-33053, London UK)
• HyperLadder V (Bioline, cat. # BIO-33057, London UK)
• L-glutamine (Sigma-Aldrich, cat. # G7513, Dorset UK)
• N2 serum free supplement (Invitrogen, cat. # 17502-048, Paisley UK)
• Non-essential amino acids (Invitrogen, cat. # 11140-035, Paisley UK)
• NT4 (Peprotech, cat. # 450-04, London UK)
• Packaging vector psPAX2 (Addgene, cat. # 12260, Middlesex UK)
• Phenol:Chloroform:Isoamyl alcohol 25:24:1 (Sigma-Aldrich, cat. # P3803, Dorset UK)
• Primers (synthesised by Sigma-Aldrich, Dorset UK) specific to TRC shRNA plasmids for lentiviral absolute quantification: forward, 5’-GGGGATTTGGGGTTGCTCTG-3’ and reverse, 5’-TTCTCTGTCCCACTCCATCCA-3’Primers (synthesised by Sigma-Aldrich, Dorset UK) specific to target and housekeeping genes for knockdown analysis
• Puromycin (Gibco, cat. # A11138-03, Paisley UK)
• Qiagen plasmid maxi kit (cat. # 12162, Manchester UK)
• Qiagen QIAamp viral RNA kit (cat. #52904, Manchester UK)
• Random hexamers (Qiagen 79236, Manchester UK)
• RQ1 DNase (Promega, cat. #M198A, Southampton UK)
• RQ stop buffer (Promega, cat. # M199A, Southampton UK)
• Sodium acetate (Sigma-Aldrich, cat. # S5636, Dorset UK)
• SuperScript III enzyme (Invitrogen, cat. # 18080-044, Paisley UK)
• SYBR Green JumpStart Taq ReadyMix (Sigma-Aldrich, cat. # S4438, Dorset UK)
• Terrific Broth medium (Sigma-Aldrich, cat. # T0918, Dorset UK)
• TriZOL reagent (Life Technologies, cat. # 15596026, Paisley UK)
• Trypsin (Sigma-Aldrich, cat. # T4174, Dorset UK)

### Recipes

• HEK medium: DMEM:F12 supplemented with 2 mM L-glutamine and 10% (v/v) FBS
• hESC medium: DMEM:F12 supplemented with 2 mM L-glutamine, 0.1% (w/v) BSA, 1× non-essential amino acids, 0.1 mM β-mercaptoethanol, 1 × N2, 1 × B27, 40 ng/ml FGF2, 10 ng/ml Activin A and 4 ng/ml NT4 [9]
• Reverse transcription (RT): Three steps: 25°C for 5 min, 50°C for 60 min and 70°C for 15 min.
• Real-time qPCR: Amplification mixtures: 5 μl of cDNA, viral cDNA or purified linearized plasmid, 10 µl of 2 × SYBR Green JumpStart Taq ReadyMix, 3 µl of distilled nuclease-free water and 1 µl of each primer at 10 µM (final concentration of 200 nM per reaction). Reaction steps: (1) initial denaturation for 3 min at 95°C; (2) 40 cycles of two-step PCR (denaturation at 95°C for 10 s, annealing and elongation at 60°C for 30 s); and (3) dissociation curve (0.5°C temperature increase every 5 s, from 65°C to 95°C).

### Equipment

• −20°C freezer
• −80°C freezer
• Bio-Rad CFX Connect machine and Bio-Rad CFX Manager 3.1 software (or any qPCR instrument and software)
• Cell incubator
• Centrifuge
• Nanodrop 2000 (Thermo Scientific, Hemel Hempstead UK)
• Shaking incubator for bacteria culture

## PROCEDURE

### shRNA plasmids: production and optimization as a qPCR standard

**Figure 2.**
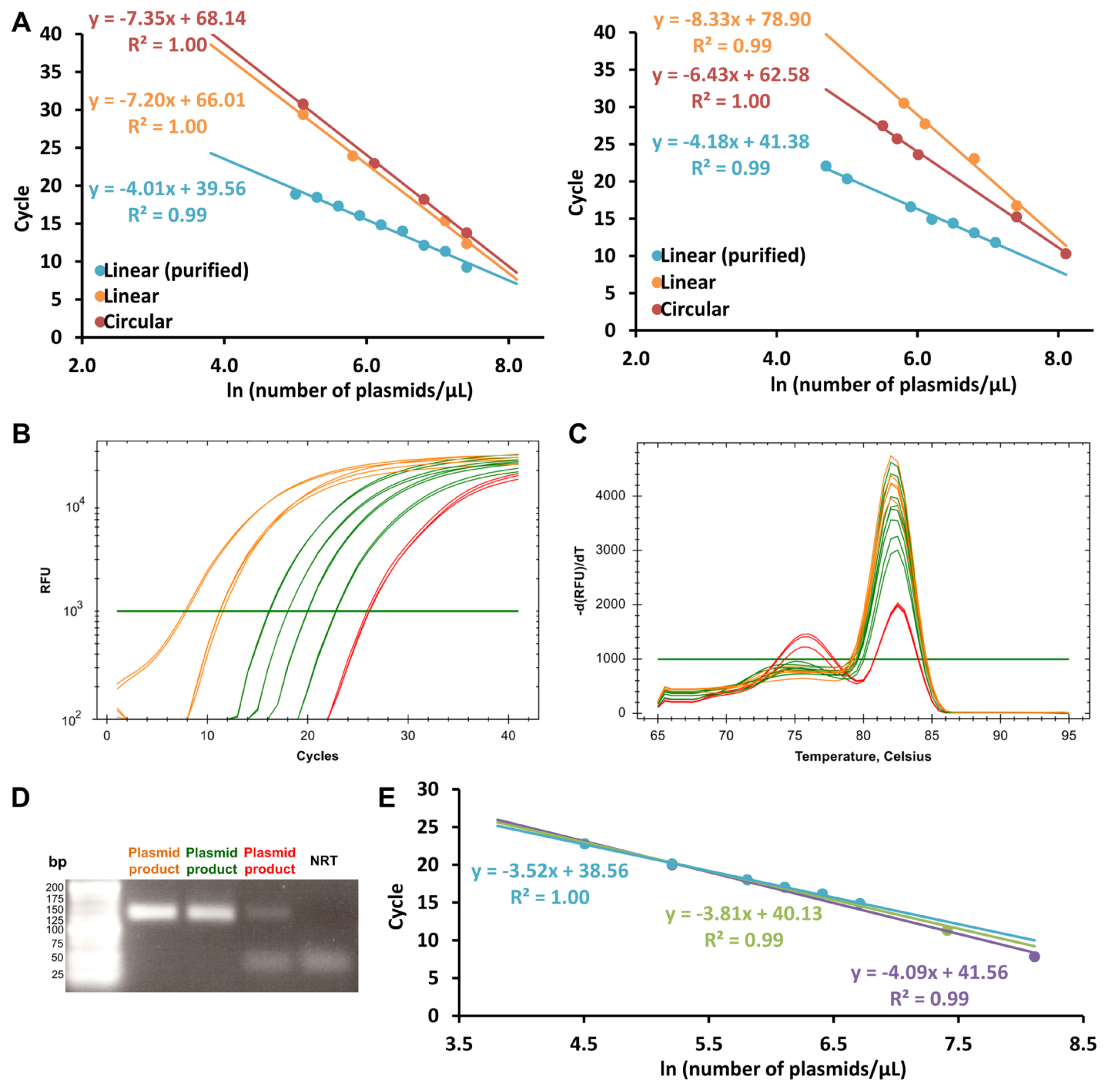
**qPCR efficiencies and dynamic range of the standard curves. A.** Efficiency curves of circular (red), linearized (orange) and purified linearized (blue): shOCT4-79 (left panel) and shControl plasmids (right panel). Linear regression curves are given with their correlation coefficient R^2^. The purified linearized plasmids give a slope of -4.01 and -4.18 respectively, which are the closest to -3.32, the ideal slope resulting in an amplification efficiency of 100%. Examples of amplification curves (B), melting peaks (C) and agarose gel of qPCR products from the shSERPINE1 (D) purified linearized standard. The tested plasmid concentration range is represented: the highest in orange, the intermediate in green and the lowest in red. **E.** Efficiency curves of purified linearized shSERPINE1 standard with the two highest plasmid concentrations (purple), without the highest concentration of 5 ng/reaction (green) or without the both highest tested concentrations of 5 and 1 ng/reaction (blue). Linear regression curves are given with their correlation coefficient R^2^. The blue curve gives a slope of -3.52 which is the closest to -3.32, the ideal slope resulting in an amplification efficiency of 100%.

1. Plasmid production
  1.1. Culture *E. coli* containing TRC shRNA plasmids overnight in a shaking incubator at 37°C in Terrific Broth with ampicillin (100 µg/ml) for selection.
  1.2. Purify plasmids using Qiagen plasmid maxi kit and dilute in 200 μl distilled nuclease-free water.
  1.3. Measure plasmid concentration and purity using Nanodrop 2000.
**NOTE**: This step is unnecessary if you buy TRC transfer vectors instead of bacterial strains.
2. Plasmid optimization as a real-time qPCR standard
**NOTE**: Other types of standards can also be used such as PCR products [10].
  2.1. Plasmid digestion
    2.1.1. Digest 500 ng of each plasmid with 1.5 units of EcoR1 for 1 h at 37°C (final volume 25 µl).
    2.1.2. Inactivate the enzyme for 20 min at 65°C.
    2.1.3. Determine digestion efficacy on a 1% (w/v) agarose gel with the HyperLadder I as molecular size marker.
  2.2. Plasmid purification
    2.2.1. Extraction: add nuclease-free water to the digested plasmids (final volume 100 µl) and one volume of phenol:chloroform:isoamyl alcohol. Vortex and centrifuge (15000 g, 2 min) at room temperature. Collect the upper aqueous phase containing the nucleic acids into a clean tube.
    2.2.2. Precipitation: precipitate plasmids by adding 2.5 × volumes of pure ethanol and 1:10 volume of 3 M sodium acetate pH 5.2. Briefly vortex and leave the solution at −80°C for at least 30 min. Centrifuge (15000 *g*, 25 min) at 4°C. Discard the supernatant and wash the pellet with 100 µl 70% (v/v) ethanol. Centrifuge (15000 g, 10 min) at room temperature. Remove the supernatant, air-dry the pellet and resuspend in the appropriate amount of nuclease-free water (usually 10−20 µl).
**NOTE**: Three different standard states (different conformations and different purities) were tested: circular plasmids, plasmids linearized by EcoR1 digestion and purified linearized plasmids. The results show that only the latter gave a reliable and efficient standard (**Fig. 2**).
3. Store plasmids at Ȣ20°C.
**TIP**: To ensure reproducibility, we advise the determination of standard stability during storage as time and temperature can significantly affect quantification [10].

### Pseudotyped lentiviral particle production

4. HEK-293TN transfection:
  4.1. Seed HEK-293TN cells at 40% confluence (4 × 10^4^ cells/cm²) in 6 well-plates with 2 ml of HEK medium for 6 h at 37°C in a humidified incubator with 5% (v/v) CO_2_.
  4.2. Change cell medium.
  4.3. Transiently co-transfect cells using Sigma Calcium Phosphate Transfection kit by adding plasmids of the second-generation packaging system at 2 µg/well and at a ratio of 4:2:1 of TRC transfer vector [1] (**Table S1**): Packaging vector psPAX2: Envelope vector pM2D.G.
**CAUTION**: Manipulating the amount of plasmids (transfer, packaging or envelope) can affect the non-functional titer without altering the functional one [11].
  4.4. Incubate cells at 37°C in a humidified incubator, 5% (v/v) CO_2_, for 16 h.
  4.5. Change cell medium.
  4.6. Incubate cells at 37°C in a humidified incubator, 5% (v/v) CO_2_, for 48 h.
5. Harvesting supernatant:
  5.1. Clear cell medium (containing the secreted pseudotyped lentiviruses) of cell debris by low-speed centrifugation (500 g, 10 min) and filter (0.45 μm filters).
  5.2. Freeze cell medium aliquots in crushed dry ice.
  5.3. Stored at −80°C.
**NOTE**: Medium from non-transfected HEK-293TN cells was used as a negative control for absolute quantification of the pseudotyped lentiviruses.

### Viral RNA extraction, cDNA synthesis and real-time PCR

6. Viral RNA extraction: purify viral RNA from cell medium using Qiagen QIAamp viral RNA kit.
7. cDNA synthesis
  7.1. Measure viral RNA concentration using Nanodrop 2000.
**NOTE**: To verify if the viral RNA was contaminated by RNAs from producer cells, we performed a viral RNA extraction on non-transfected HEK-293TN cells. RNAs were detected by Nanodrop 2000 measurement, suggesting that exosomes/vesicles containing RNAs were secreted by the HEK-293TN cells [12].
  7.2. Treat viral RNA (500 ng) with 1 unit of RQ1 DNase for 30 min.
**NOTE**: Because HEK-293TN cells were transiently transfected, transfer vector could contaminate the pseudotyped lentivirus supernatant. To avoid the amplification of contaminating plasmid DNA, viral RNA was DNase-treated prior to RT. This step could be omitted if a stable producer cell line was used instead [13]. DNase-treated viral RNA was used as qPCR non-RT (NRT) control to ensure no remaining contamination from plasmid DNA.
  7.3. Perform RT according to the supplier instruction by adding 100 ng of DNAfree viral RNA with 200 units of SuperScript III enzyme, 200 ng of random hexamers and 10 mM of dNTPs. Assume the reaction to be linear and 100% efficient [14].
**CAUTION**: Use random hexamers and not oligo-dTs, which are unable to bind to viral RNAs due to the absence of poly A tails [15].
**CAUTION/TIP**: RT efficiency can be impacted by different factors such as the type of RT enzyme used [16,17], the amount of RNA [17] or its quality (*i.e.*, integrity [18,19] and purity [17]).
  7.4. Store viral RNA and cDNA at −80°C.
**CAUTION**: Storage conditions impact RNA integrity, and therefore, downstream analysis [19].
8. Real-time qPCR:
  8.1. Perform real-time qPCR on a qPCR instrument.
**NOTE**: The pair of primers used to amplify the 124 base pair sequence (**Fig. S1**) was tested for sequence complementarity against the following human and mouse databases: RefSeq mRNA, Genome, NCBI Chromosome Sequences, Transcript Reference Sequences and Nucleotide Collection. As expected, they were highly specific to the HIV-1 integration site (data not shown). Other predicted PCR products were too large to be amplified by our qPCR reaction conditions (data not shown).
**NOTE**: Primers were designed against the sequence element of the shRNA plasmid that is integrated into the host genome after transduction. Specifically, they are located immediately following the Rev response element sequence (**Fig. S1**). Even though this was not within the scope of our research, these primers can be potentially used for functional titration of both integrated proviral DNA copies and expressed transgene before complete splicing, overcoming the fact that the shRNA plasmids do not contain a fluorescent reporter gene [1].
  8.2. Perform qPCR in triplicate using a range of different plasmid concentrations.
**NOTE**: 12 concentrations were tested, from 0.3 pg/reaction to 5 ng/reaction.
**CAUTION**: The workable concentration range of the standard plasmid is an important parameter: very high template concentrations can interfere with the qPCR reaction and decrease its efficiency (E), whereas very low template concentrations can result in primer dimer formation.
  8.3. Perform qPCR in triplicate using serial dilutions of viral cDNA from untransfected (negative control) and transfected HEK-293TN.
**NOTE**: 5 concentrations were tested, from 0.6 to 7.5 ng/reaction (2 × serial dilution).
**CAUTION**: The workable concentration range for qPCR is a key parameter: low viral cDNA concentrations tend to overestimate the absolute number of pseudotyped lentiviruses due to the presence of primer dimers, whereas high viral cDNA concentrations can inhibit the PCR, resulting in the number of lentiviruses being underestimated.
  8.4. Perform ‘no reverse transcription’ (NRT) and ‘no template controls’ (NTC) to confirm absence of contamination.
  8.5. Run qPCR products on a 2.5% (w/v) agarose gel with the HyperLadder V as molecular size marker to verify product sizes.
**NOTE**: qPCR product specificity is validated by comparing qPCR product melting peak and gel electrophoresis of the viral cDNA templates with NRT, NTC and negative controls.

### Lentiviral particle absolute quantification

9. Construct standard curves
  9.1. Use purified linearized plasmids as standards for absolute quantification.
  9.2. Extract raw qPCR data.
  9.3. Use the concentrations yielding the best technical replicates in terms of their similarity of signal and the absence of primer dimers (checked using melting peak data) to generate standard curves.
10. Determine qPCR amplification efficiencies (E)
  10.1. Plot cycle threshold (Ct) values of the standard dilutions against the logarithm of N (the number of single-stranded plasmid molecules/μl) calculated as follows [20]:

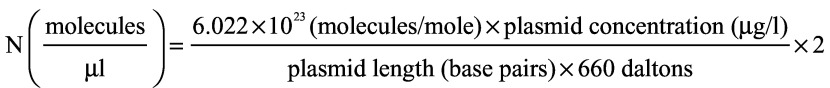

**NOTE**: A factor of 2 is applied to N to take into account that plasmids are double-stranded while viral RNA and cDNA are single-stranded.
  10.2. Fit linear regression curves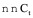 to establish 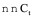 [21]. A slope of -3.32 is equivalent to E = 1 (or 100%).
11. Use the same standard curves to establish the pseudotyped lentiviral particle absolute titer as follows:

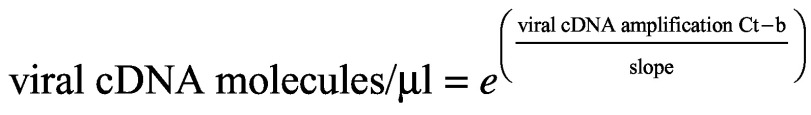

And

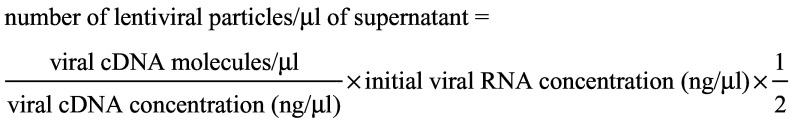

**NOTE**: Each lentivirus carries two RNA copies implying a theoretical ratio of ½ [22].
**TIP**: To better estimate the absolute titer, more than one cDNA concentration was utilized to calculate the final quantification.

### Cell culture, transduction and knockdown analysis

**NOTE**: This section uses hESCs as a model cell line but other cell lines could be used instead.

12. Cell culture:
  12.1. Coat culture dishes with 100 μl/cm^2^ fibronectin at 25 μg/ml for 1 h at 37°C.
  12.2. Culture the hESCs in a serum-free and feeder cell-free preparation developed in our laboratory [9] and incubate them at 37°C in a humidified incubator with 5% (v/v) CO_2_.
**NOTE**: Other hESC growth medium may be used.
  12.3. Change culture medium every two days.
  12.4. Passage cells every three to five days by trypsinization (50 μl of 0.5 × trypsin/cm^2^).
13. Cell transduction
At day 0:
  13.1. Seed hESCs at 2600 cells/cm^2^.
  13.2. Incubate cells at 37°C in a humidified incubator, 5% (v/v) CO_2_, for 6 h.
  13.3. Add lentiviruses to the cell medium.
**NOTE**: Concentrations of 18 pg viral RNA/cell (C1) and 36 pg viral RNA/cell (C2) were used in the present study.
**CAUTION**: For different shRNAs, equivalent amounts of extracellular RNA may not equate to equivalent concentrations of the respective lentiviruses.
At day 1:
  13.4. Change cell medium.
  13.5. Incubate cells for two more days.
At day 3:
  13.6. Passage cells.
  13.7. Add puromycin to the medium at a concentration of 0.5 μg/ml for selection.
At day 5:
  13.8. Lyse cells for RNA extraction using TriZOL reagent.
  13.9. Store lysates at −80°C or perform RNA extraction (see the following section).
14. Knockdown analysis
  14.1. RNA extraction
    14.1.1. Purify RNA by chloroform extraction (1:5 of TriZOL volume, 10 min incubation at room temperature, 15 min centrifugation at 12000 g, 4°C) and isopropanol precipitation (1:2 of TriZOL volume added to the aqueous phase, 10 min incubation at room temperature, 15 min centrifugation at 12000 g, 4°C).
    14.1.2. Wash RNA pellets with 75% (v/v) ethanol (1:1 of TriZOL volume, 15 min centrifugation at 12000 g, 4°C).
    14.1.3. Dissolve RNA pellets in 25 to 100 µl of distilled nuclease-free water.
    14.1.4. Measure RNA concentration and purity using Nanodrop 2000.
    14.1.5. Store RNAs at −80°C.
**CAUTION**: Storage conditions impact RNA integrity and, therefore, downstream analysis [19].
  14.2. cDNA synthesis:
    14.2.1. Treat RNA (1 µg) with 1 unit of RQ1 DNase for 30 min.
    14.2.2. Stop the reaction by adding 1 µl of RQ stop buffer followed by a 15-min incubation at 60°C.
    14.2.3. Perform RT according to the supplier instruction by adding 200 ng of DNase-treated RNA with 200 units of SuperScript III enzyme, 200 ng of random hexamers and 10 mM of dNTPs. Assume the reaction to be linear and 100% efficient [14].
**CAUTION/TIP**: RT efficiency can be impacted by different factors such as the type of RT enzymes used [16,17], the amount of RNA [17], RNA quality (integrity [18,19] and purity [17]) and the priming strategy [23].
    14.2.4. Store cDNA at −80°C.
  14.3. Real-time qPCR:
    14.3.1. Design PCR primers with NCBI primer-BLAST online tool [24] to amplify all variant transcripts of each target gene with the following parameters: PCR product size between 60 and 150 base pairs, melting temperature (Tm) at 60.0°C ± 1.0°C with a maximum Tm difference of 1.0°C and primers spanning an exon-exon junction or being separated by at least one intron if possible.
    14.2.2. Perform real-time qPCR on target and housekeeping genes. Run each sample in triplicate.
**NOTE**: In the present study, target genes were neuropilin 1 (NRP1), plexin B1 (PLXNB1) and octamer-binding transcription factor 4 (OCT4, protein encoded by Pit-1 octamer unc-86 class 5 homeobox 1, abbreviated Pou5f1), and housekeeping genes were HPRT1, MAPK1 and UBC (**Table S2**).
CAUTION:
• qPCR primer efficiency should be assessed [25].
• Housekeeping gene stability should be assessed and validated by a coefficient of variance < 0.5 and the M-value < 1 [26].
• Each qPCR plate should contain a calibrator (two wells with the same cDNA in each plate) to mitigate plate-to-plate variability in the analysis.
• NRT and NTC should be performed to confirm absence of contamination.
• If the C_t_ standard error of the mean (SEM) is > 0.25, the outlier C_t_ should be removed from the analysis, or the sample should be rerun.
  14.4. Relative quantification: extract raw qPCR data and calculate the relative quantification using the method [27].
**NOTE**: Raw qPCR data were extracted and analyzed with Bio-Rad CFX Manager 3.1 software in the present study. Statistical analysis was performed using IBM SPSS Statistics 21 software.
  14.5. Correlation between knockdown and shRNA lentiviral concentration:
    14.5.1. Convert viral RNA concentration into number of viral particles if need be.
    14.5.2. Plot the knockdown percentage as a function of the absolute number of viral particles (plot each target gene independently).
    14.5.3. Use a logarithmic fitting curve to describe the correlation.

## ANTICIPATED RESULTS

### Optimization of the qPCR standard

To obtain a precise and accurate measurement of the absolute titer of pseudotyped lentiviral particles, real-time qPCR standards have to be as reliable and efficient as possible. According to our results, when using plasmids as standard, the purified linearized form was the most appropriate, with a qPCR efficiency (E) of 84% and 73% in the given examples (**Fig. 2A** and **2B**). For circular plasmids, E was 37% and 43%, suggesting that the circular shape had a negative effect on the PCR reaction. The PCR reaction was also inhibited when the linearized plasmid was not purified (E = 38% and 32%).

The workable concentration range for qPCR also needs to be established by: (1) verifying the absence of primer dimers (especially for the low cDNA concentrations, from 0.3 pg/μl in the present case) using qPCR product melting peaks (**Fig. 2C**) and gel electrophoresis (**Fig. 2D**); (2) checking if the highest cDNA concentration(s) lower E. Our data demonstrated that removing the two highest tested plasmid concentrations of 5 ng/µl and 1 ng/μl improved E by 19% ± 8% (**Fig. 2B-2E, Table 1, Fig. S2**).

### Specificity of the real-time qPCR

**Figure 3.**
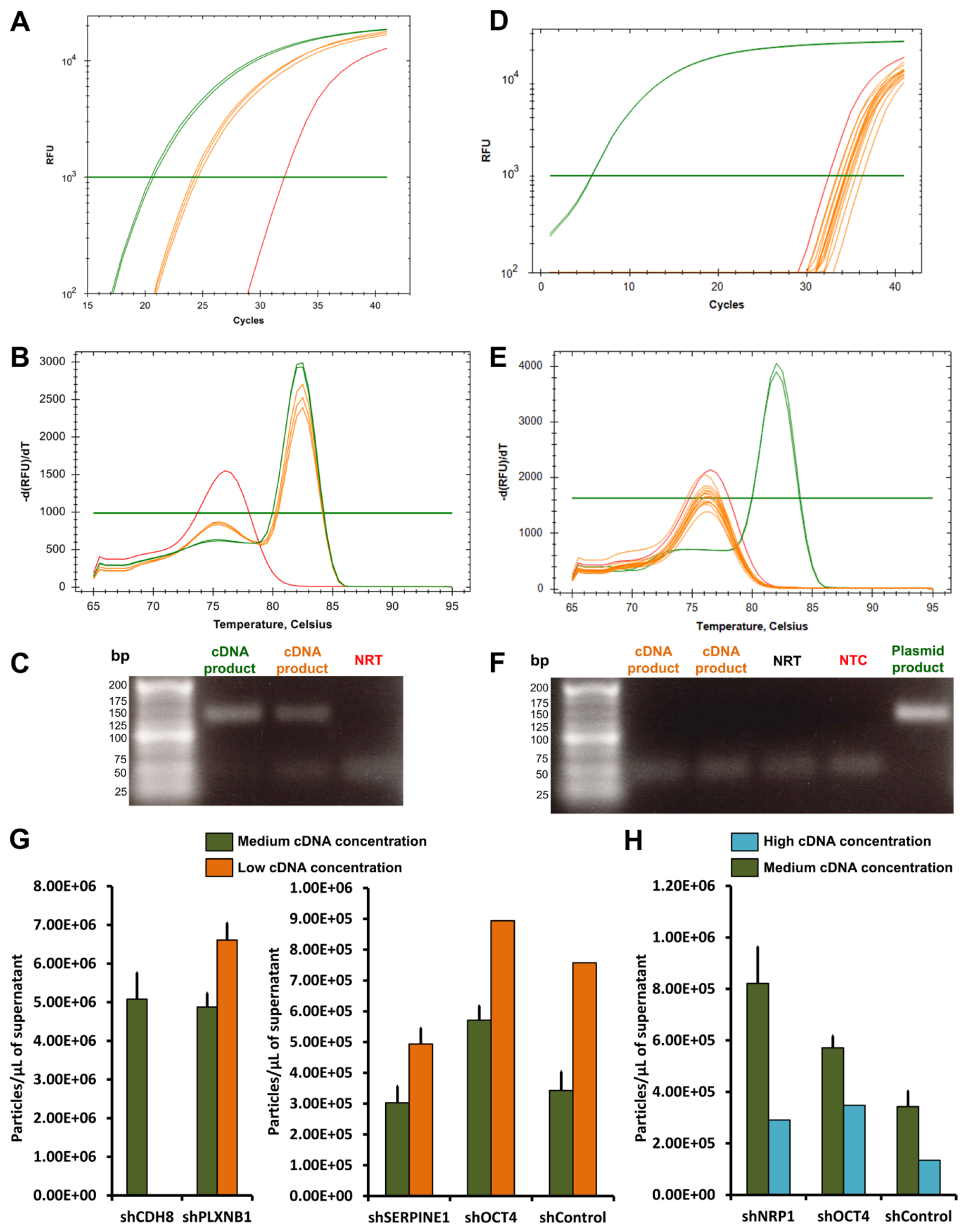
**shRNA pseudotyped lentiviral particle absolute quantification. A-C.** Viral cDNA quantification by qPCR: examples of amplification curves (A), melting peaks (B) and agarose gel of qPCR products from the shPLXNB1 viral cDNA (C). The viral cDNA concentration in green was the highest tested and in orange the lowest. The NRT is represented in red. **D-F.** cDNA amplification from non-transfected producer cells: amplification curves (D), melt peaks (E) and agarose gel of qPCR products from amplified cDNA of purified secreted RNA of HEK-293TN cells in orange, an example of amplified shOCT4-79 plasmid in green and NTC in red (F). **G-H.** Effect of the lowest and highest viral cDNA concentrations on shRNA pseudotyped lentiviral particle absolute quantification: comparisons of shRNA lentiviral particle absolute quantification using intermediate (medium) and the lowest (G) or the highest (H) viral cDNA concentrations (pseudotyped lentiviral particles/µl of supernatant, mean ± SEM; shNRP1=shNRP1-24; shOCT4=shOCT4-79; shPLXNB1=shPLXNB1-35).

Viral RNA should be DNase-treated prior to RT to avoid quantification bias due to contaminant plasmids from transfected HEK-293TN cell medium. The real-time qPCR must contain the following controls: (1) cDNA from viral RNA of untransfected HEK-293TN cell medium as negative control; (2) DNase-treated viral RNA as NRT and (3) NTC. The qPCR melting peaks, as well as gel electrophoresis of the controls were compared to those from samples for assessing product specificity (**Fig. 3A-3F**). None of the tested cDNA concentrations from non-transfected HEK-293TN cells gave a specific amplification of our product of interest (**Fig. 3D-3F**).

**Table 1.** Standard efficiencies.

	shControl	shCDH8	shNRP1-24	shOCT4-79	shPLXNB1-35	shSERPINE1	Mean	SEM
A	0.63	0.79	0.75	0.64	0.70	0.75	0.71	0.06
B	0.76	0.88	0.85	0.71	0.75	0.83	0.80	0.07
C	0.78	0.99	0.91	0.99	0.82	0.92	0.90	0.09

Calculated efficiencies of six different purified and linearized shRNA plasmids. A: with the both highest tested concentrations; B: without the highest concentration of 5 ng/reaction; C: without the both highest tested concentrations of 5 and 1 ng/reaction.

**Figure 4.**
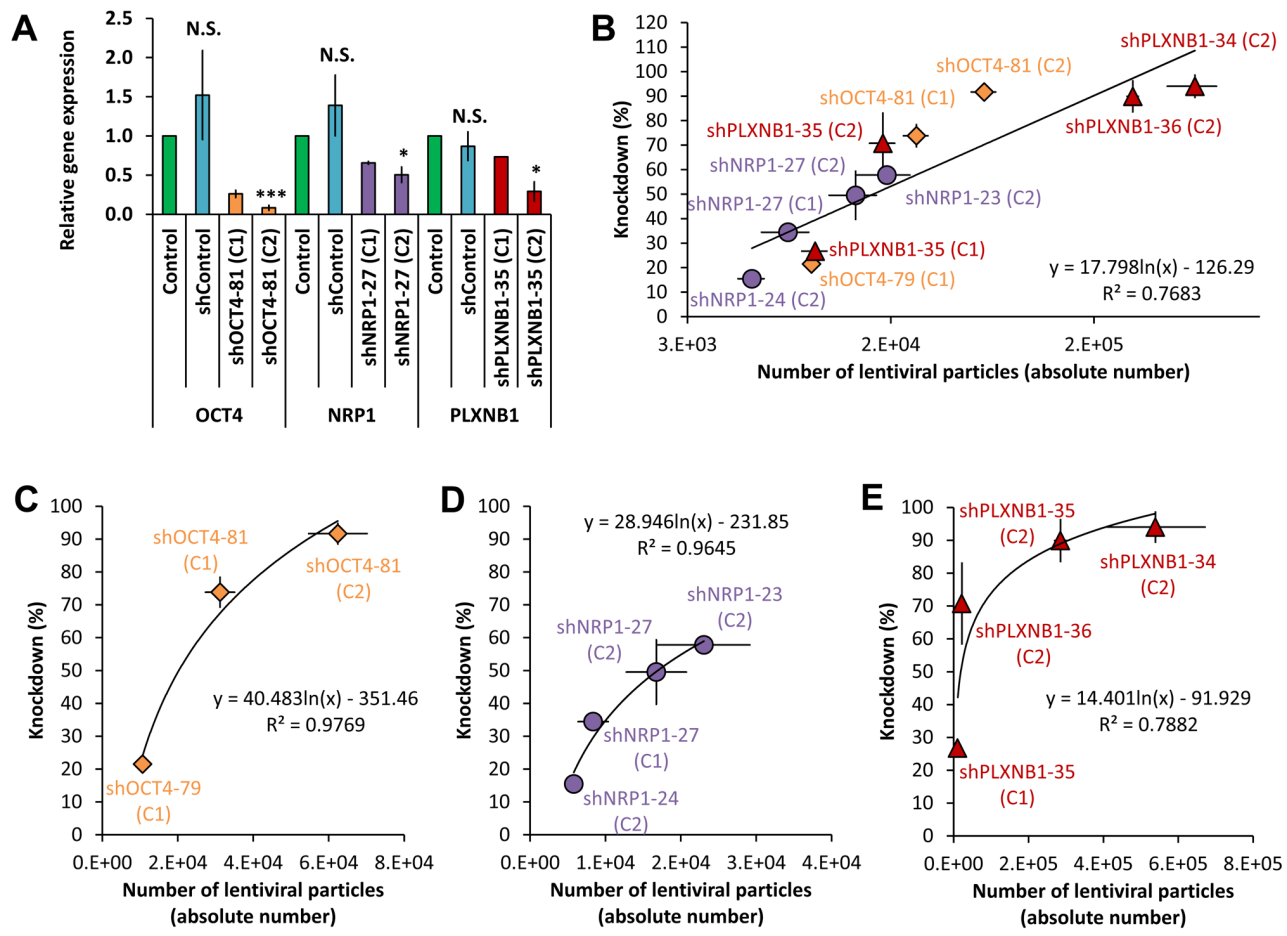
**Effect of shRNA lentiviral concentration on knockdown efficiency. A.** Relative gene expression results measuring the knockdown efficiency of NRP1, PLXNB1 and OCT4 with two different shRNA concentrations C1 (18 pg viral RNA/cell) and C2 (36 pg viral RNA/cell. Only C2 was used for shControl ( normalisation done against untransduced hESCs; mean ± SEM; One sample *t*-test, *0.01 < *P* < 0.05, *** *P* < 0.001, N.S.: not significant, no star means n < 3). **B-E.** Correlations (logarithmic fitting curves) between the amount of shRNA lentiviral particles (absolute number) and the knockdown efficiency (%) of NRP1 (purple), PLXNB1 (red) and OCT4 (orange) together or separately.

### Absolute quantification of the pseudotyped lentiviral particle

Similarly to the plasmids, our data demonstrate that it is very important to determine the workable concentration range suitable for viral cDNA amplification. Low viral cDNA concentrations resulted in non-specific amplification of primer dimers (**Fig. 3A-3C**), leading to an overestimation of the absolute number of pseudotyped lentiviral particles (**Fig. 3G**). High viral cDNA concentration led to PCR reaction inhibition and, therefore, to the underestimation of the absolute number of pseudotyped lentiviral particles (**Fig. 3H**). Because the suitable concentration range for titration was limited (usually between 1 and 7.5 ng/reaction maximum), calculating the cDNA amplification efficiency was not relevant.

### shRNA lentiviral concentration and knockdown, a correlation

The highest viral RNA concentration triggered higher knockdowns (**Fig. 4A**), but no correlation was apparent between RNA concentration and knockdown efficacy. This may be due to the measurement of lentiviral shRNA concentration in terms of extracellular RNA, which may be confounded by contributions from cellular RNAs. Thus, for different shRNAs, equivalent amounts of extracellular RNA may not equate to equivalent concentrations of the respective lentiviruses. In contrast, when the conversion into absolute number of viral particles was done using the method described above (**Fig. 1**), there was a positive correlation with the knockdown efficacy (Fig. 4B). Target genes were expressed at different levels in hESCs in terms of qPCR Ct range: OCT4 (21.5 < Ct < 26.5, control cells; 26 < Ct < 39, knockdown cells) was more highly expressed than NRP1 (27.5 < Ct < 31.5, control cells; 27 < Ct < 33, knockdown cells), which was highly expressed than PLXNB1 (30.5 < Ct < 36.5, control cells; 33 < Ct < 39, knockdown cells). Consequently, the correlation was stronger when each gene was analyzed individually (**Fig. 4C-4E**). The differences in knockdown efficiency might be linked to the expression level of each gene [28] and/or to the shRNA sequences themselves [29]. Moreover, the correlation seemed to be more logarithmic than linear, which is consistent with published results of expressed transgene quantification by RT-qPCR in mesenchymal stem cells [30].

## TROUBLESHOOTING

**Table 2** recapitulates the main sensitive points for achieving a reliable absolute quantification of produced lentiviral particles.

**Table 2.** Troubleshooting table.

Step	Sensitive point(s)
Optimization of the real-time qPCR standard	If using a plasmid as standard, prefer the purified linearized state
	Run agarose gels to verify plasmid digestion Determine good conditions for the standard storage (over time and temperature) [10]
	Determine the workable concentration range for qPCR (Too high = decrease of E; Too low = formation of primer dimers)
Pseudotyped lentiviral particle production	Manipulating the amount of any plasmid (transfer, packaging or envelope) can affect the non-functional titer without altering the functional one [11]
Specificity of the real-time qPCR	Viral RNA purification: Perform a viral RNA extraction on non-transfected HEK-293TN cells to use as a negative control Do a DNase treatment of the pseudotyped lentiviral particle supernatant prior to reverse transcription to degrade potential plasmid left-overs during transient transfection
	Real-time qPCR: If using a novel pair of primers, test their complementary against suitable databases (Refseq mRNA, *etc.*) Use suitable qPCR controls (*e.g.* NRT, NTC*) Construct the qPCR dissociation curves Run agarose gels to verify qPCR product sizes
Pseudotyped lentiviral particle absolute quantification	Determine the workable concentration range for qPCR (Too high = PCR inhibition and decrease of E, leads to an underestimation of the absolute number of produced particles;
	Too low = formation of primer dimers, leads to an overestimation of the absolute number of produced particles) Use more than 1 viral cDNA concentration for calculation
Cell culture, transduction and knockdown analysis	Prefer the use of absolute number of viral particles as metrics rather than viral RNA concentration to standardize knockdown experiments

*NRT: no reverse transcription; NTC: no template controls.

## CONCLUSIONS

Many studies have demonstrated that shRNA lentiviral-based approaches provide efficient and stable knockdown in various cell types. The present guide is a simple way to non-functionally quantify the absolute number of produced pseudotyped TRC shRNA lentiviral particles in a batch. The quantification can be used prior to transduction for protocol standardization and optimization, such as finding the best producer cell line or investigating the optimal harvesting period. Moreover, this non-functional titration rapidly provides insight into the knockdown efficacy that can be achieved, without doing a functional titration. The same primer pair can potentially be used for functional titration of both the integrated proviral DNA copies and the expressed transgene level in mouse and human cells.

## Abbreviations used

E: amplification efficiencies
BSA: bovine serum albumin
Ct: cycle threshold
FBS: fetal bovine serum
HEK: human embryonic kidney
hESCs: human embryonic stem cells
Tm: melting temperature
NRP1: neuropilin 1
NRT: no reverse transcription
NTC: no template controls
OCT4: octamer-binding transcription factor 4
PLXNB1: plexin B1
qPCR: quantitative PCR
RT: reverse transcription
RNAi: RNA interference
shRNA: short hairpin RNA
SEM: standard error of the mean
TRC: the RNAi Consortium

## Supplementary information

**Table S1**. shRNA plasmid references and sequences.

**Table S2**. PCR primer references and sequences.

**Figure S1**. Partial pLKO.1-puro plasmid sequence.

**Figure S2**. Effect of the highest standard concentrations on qPCR efficiencies.
